# Two novel alleles in *C. elegans* *mir-1822* gene.

**DOI:** 10.17912/micropub.biology.000212

**Published:** 2020-01-16

**Authors:** Marcy Anderson, Maddy Mash, Dustin Haskell, Shilpa Hebbar, Anna Y Zinovyeva

**Affiliations:** 1 Division of Biology, Kansas State University, Manhattan, KS, USA

**Figure 1.**
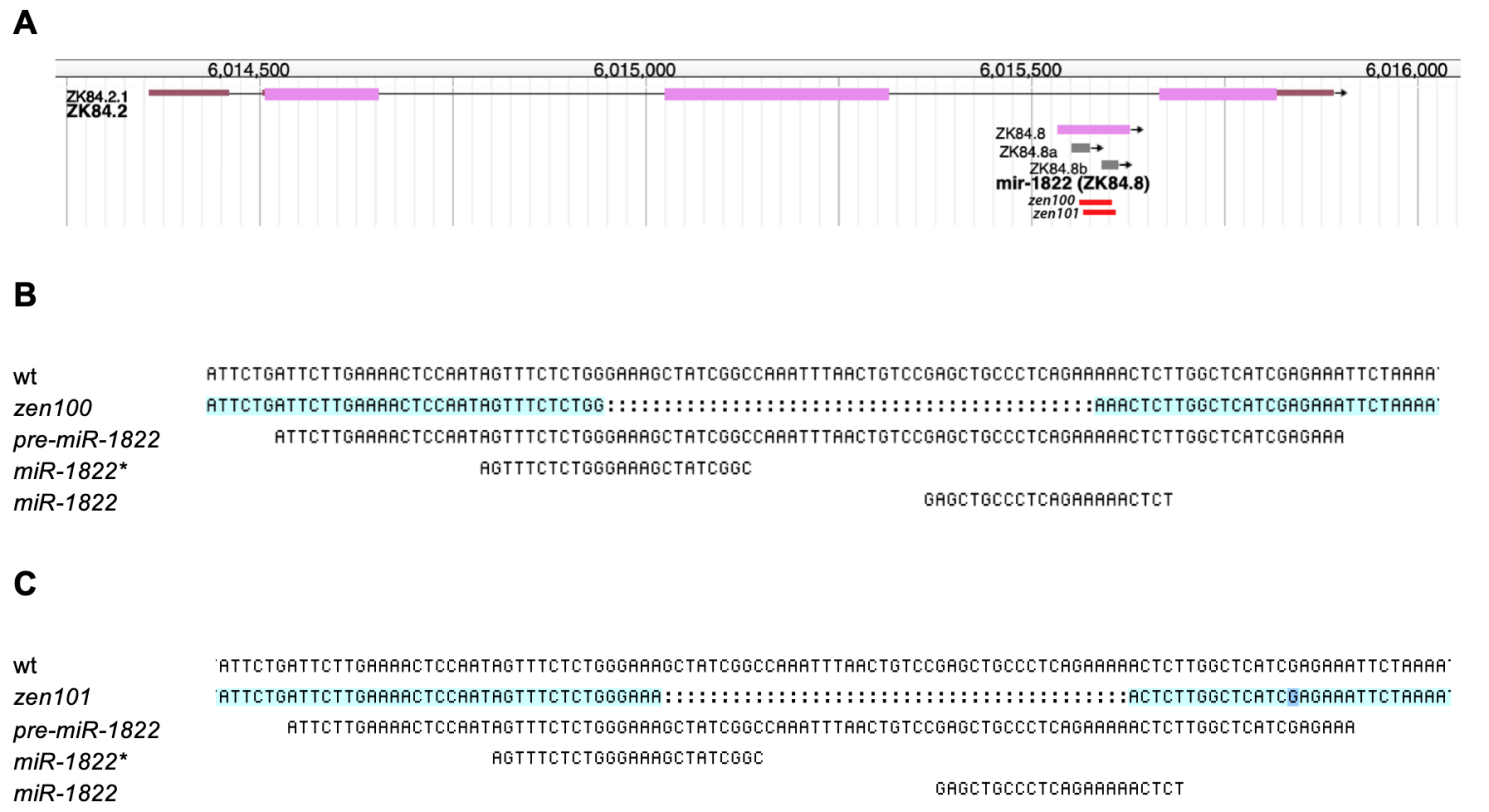
(A) *zen100* and *zen101* are novel alleles of *mir-1822*. (B,C) Sequence information for the two *mir-1822* deletions. (B) *zen100* removes 43 bps from the *mir-1822* locus. (C) *zen101* removes 41 bps from the *mir-1822* locus.

## Description

microRNAs are small noncoding RNAs of ~22 nucleotides in length that regulate gene expression by degrading target mRNAs or inhibiting their translation. To our knowledge *mir-1822* currently lacks deletion alleles, impeding *mir-1822* functional characterization. We generated two new deletions of the *C. elegans* *mir-1822* locus, using the CRISPR-Cas9 genome editing technique. The following *mir-1822* specific Alt-R crRNAs were ordered from IDT: gRNA1, 5’-AGTTTCTCTGGGAAAGCTAT-3’ and gRNA2: 5’-TGAGCCAAGAGTTTTTCTGA-3’. To create the deletions, Cas9 (Alt-R Cas9, IDT) was loaded with the two *mir-1822* guide RNAs, *dpy-10* guide RNA (Arribere et al, 2014) (IDT), and tracer RNA (IDT) and the mixture was injected into *C. elegans*. The resulting progeny were screened for CRISPR-Cas9 positive animals as previously described (Arribere et al, 2014). The following PCR primers were used to screen for deletions of interest: mir-1822.for1: 5’- CGGAAGGACACCTGCCACCAATG-3’ and mir-1822.rev1: 5’- GAGGGCAATCTTCTTCTGGTCGCC -3’.

Using PCR screening, we identified two independent deletions of approximately 40 nucleotides each, with the positions of each deletion schematized in Figure 1A. *mir-1822(zen100)* removes 43 base pairs (Fig. 1B), and *mir-1822(zen101)* deletion removes 41 base pairs from the *mir-1822* precursor region (Fig. 1C). Each deletion was sequenced twice for confirmation. Both *mir-1822* alleles are homozygous viable and appear to be superficially wild type, with no obvious phenotypes observed in either strain.

## Reagents

UY265 *mir-1822(zen100)* and UY266 *mir-1822(zen101)* strains are available upon request.
